# Morphological changes after lower eyelid epiblepharon surgery in Asian children

**DOI:** 10.1186/s12886-021-02052-9

**Published:** 2021-08-06

**Authors:** Sang Jae Lee, Shin-Hyo Lee, Min Sub Lee, Youn Hye Jo, Hyun Jin Shin, Andrew G. Lee

**Affiliations:** 1 School of Medicine, Konkuk University, Seoul, Republic of Korea; 2 Department of Anatomy, Yonsei University College of Medicine, Seoul, Republic of Korea; 3 Department of Ophthalmology, Research Institute of Medical Science, Konkuk University Medical Center, Konkuk University School of Medicine, 120 Neungdong-ro, Gwangjin-gu, Seoul, Republic of Korea 143-701; 4 Department of Ophthalmology, Blanton Eye Institute, Houston Methodist Hospital, Houston, TX USA; 5 Department of Ophthalmology, Neurology, Neurosurgery, Weill Cornell Medicine, New York, NY USA; 6 Department of Ophthalmology, University of Texas Medical Branch, Galveston, TX USA; 7 Department of Ophthalmology, UT MD Anderson Cancer Center, Houston, TX USA; 8 Department of Ophthalmology, Texas A and M College of Medicine, College Station, TX USA; 9 Department of Ophthalmology, University of Iowa Hospitals and Clinics, Iowa City, IA USA; 10 Department of Ophthalmology, Baylor College of Medicine and the Center for Space Medicine, Houston, TX USA; 11 Department of Ophthalmology, University of Buffalo, Buffalo, NY USA

**Keywords:** Epiblepharon, Morphological change, Marginal reflex distance, Inferior half area

## Abstract

**Background:**

This study aimed to determine the morphological changes in Asian lower eyelid epiblepharon patients after surgery.

**Methods:**

The medical records of 59 patients who underwent lower eyelid epiblepharon repair were reviewed retrospectively. Eighty-nine patients who underwent strabismus surgery were set as the control group. The photographs for each group were analyzed based on the following factors: inferior half area (IHA) of the eye, eyelash angular direction (EAD), angle between the eyelashes and the cornea, marginal reflex distance 1 (MRD_1_) and marginal reflex distance 2 (MRD_2_).

**Results:**

After surgery, the medial EAD changed from 92.45° ± 20.21° (mean ± SD) to 79.43° ± 23.31°, while the central and lateral EADs were unchanged. IHA increased from 36.33 ± 9.78 mm^3^ to 43.06 ± 10.57 mm^3^, and MRD_1_ increased from 1.92 ± 0.99 mm to 2.50 ± 0.93 mm, whereas MRD_2_ did not change. The mean angle between the eyelashes and the cornea increased from 39.64° to 72.19° immediately postoperatively, but had reduced to 58.75° 3 months later, followed by no further significant change at the 6-month and 9-month postoperative follow-ups.

**Conclusions:**

There is morphological changes of the eyelid after lower eyelid epiblepharon surgery, with increases in the IHA and MRD_1._ In addition, contact between the eyelashes and the cornea occurred mainly in the medial portion of the eyelid the position, which everted and stabilized over 3 months. Thus, follow-up observations are required for at least 3 months to properly evaluate the surgical outcome.

## Background

Epiblepharon is a common eyelid anomaly in Asian children, and often involves the lower eyelids bilaterally [1, 2]. In this condition, a horizontal fold of the skin and pretarsal orbicularis override the eyelid margin, causing the eyelashes to turn inwards. Keratopathy may additionally develop because of prolonged corneal contact by the eyelashes or frequent rubbing of the eyes [3]. The symptoms in most Caucasians are known to disappear with facial bone growth, but in Asians the symptoms often persist and occasionally result in corneal opacity. This means that surgical correction is often necessary when the condition persists or is particularly serious acutely [4, 5].

The purpose of epiblepharon surgery is to improve the symptoms, focusing on preventing recurrence and avoiding side effects. Moreover, parents and patients have considerable interest in the associated morphology and sometimes are concerned about appearance changes after surgery. Several previous studies have analyzed the surgical outcome depending on the shape of the eyelid before surgery in epiblepharon patients. For example, Oh et al. reported on eyelashes changes from a lateral perspective [6]. They interpreted the postoperative improvement based on the angle between the eyelashes and the cornea, and found that successful surgical results were associated with larger angles. However, there is a lack of studies that analyzed pre- to postoperative morphological changes with in Asian children with epiblepharon.

In this study we used multidimensional and longitudinal assessments to analyze morphological changes after lower eyelid epiblepharon surgery. We compared pre- and postoperative facial images from frontal and lateral perspectives in epiblepharon patients, and also compared them with a control group without eyelid anomalies.

## Methods

This retrospective case–control study was conducted at the Department of Ophthalmology at Konkuk University Medical Center in Seoul, South Korea. The study was performed in accordance with the principles of the Declaration of Helsinki, and its protocol was approved by the institutional review board and ethics committee at Konkuk University Medical Center (IRB approval number: KUH100072). We reviewed facial images of 59 Korean children (118 lower eyelids) who underwent lower eyelid epiblepharon surgery between January 2015 and December 2017. Eighty-nine age-matched patients (178 lower eyelids) who underwent strabismus surgery without eyelid anomalies were used as controls for a comparative analysis. The following exclusion criteria were applied: (1) other eyelid problems affecting eyelid morphology, such as upper eyelid epiblepharon or ptosis, or previous eyelid surgery history, (2) > 15 years of age, and (3) postoperative follow-up shorter than 6 months.

### Surgery and postoperative follow-up

**Fig. 1:**
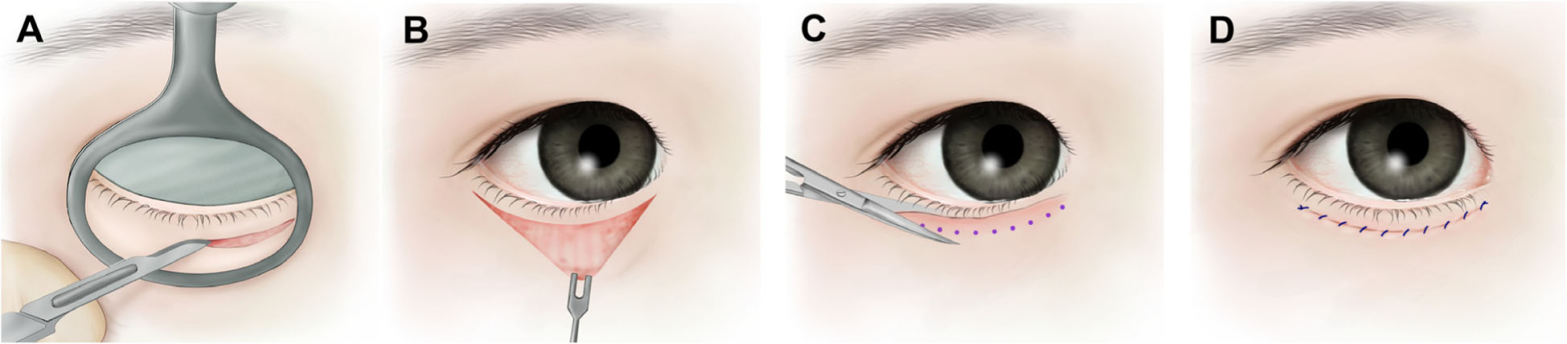
Surgical technique. **A** A large chalazion clamp is applied to the lower eyelid and stabilize the lower eyelid during the incision. **B** After subciliary incision, several buried 7–0 Vicryl sutures were placed to allow adhesion between the tarsal plate and the subcutaneous tissue of the upper skin flap. **C** Minimal amounts of redundant skin and orbicularis muscle are removed to avoid ectropion or lower eyelid retraction. **D** After meticulous haemostasis, the skin is closed with a running 6–0 fast absorbing gut suture

The lower epiblepharon surgeries were performed in a standardized fashion as described by Woo et al. [7] After subciliary incision, three buried 7–0 Vicryl sutures (Ethicon; Johnson and Johnson, Livingston, UK) were placed to allow adhesion between the tarsal plate and the subcutaneous tissue of the upper skin flap with minimal resection of the pretarsal orbicularis and redundant skin (Fig. 1). The first ciliary everting suture was placed center part of tarsus. The second suture was placed 2–3 mm laterally to the lower punctum. The third suture was placed approximately 3–4 mm laterally to the first suture. All surgeries were performed by a single surgeon (H.J.S.). Patients were examined before surgery and 1 week, 1 month, 3 months, 6 months, and 9 months after surgery.

### Measurements

**Fig. 2:**
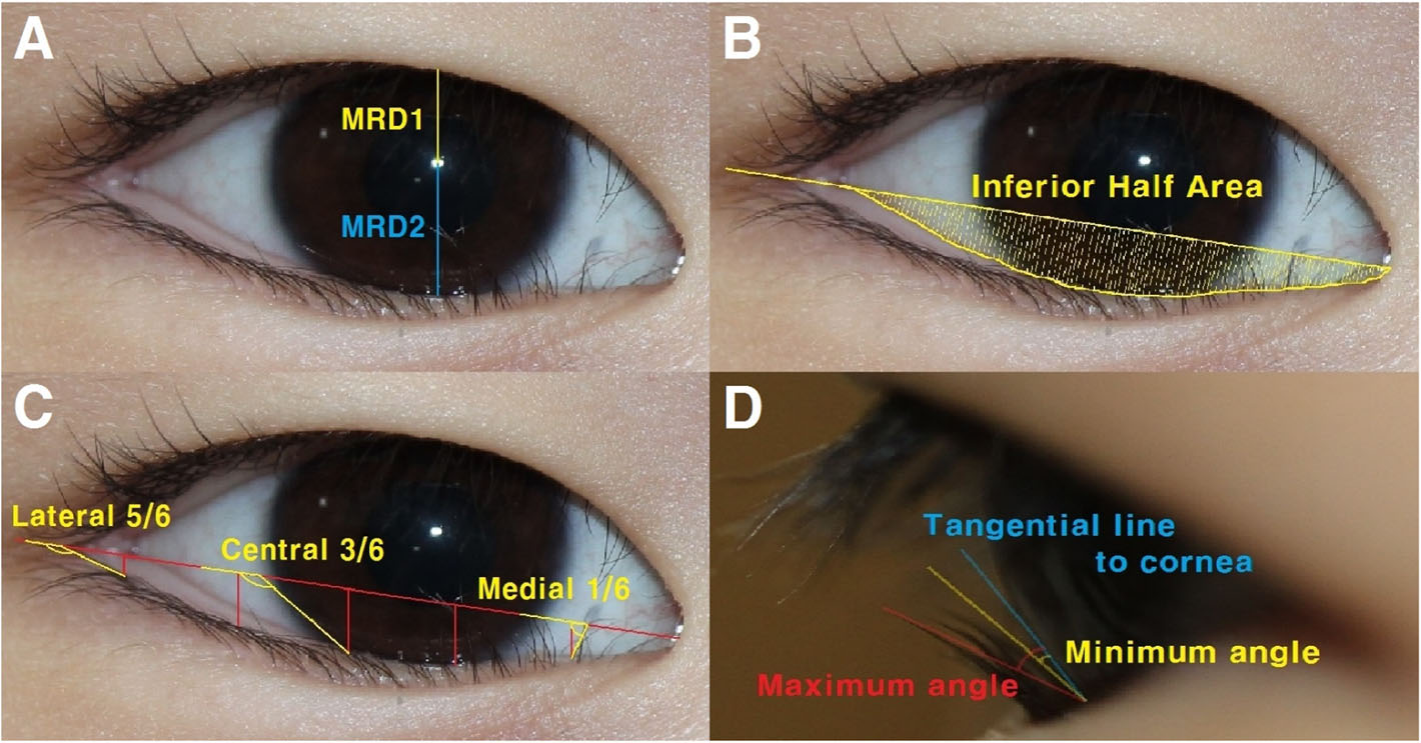
Measurements of (**A**) marginal reflex distance 1 (MRD_1_) and marginal reflex distance (MRD_2_). **B** inferior half area (IHA), **C** eyelash angular direction (EAD), **D** side-view angle (SVA), and these measurements are described in detail in the Measurements section of the Materials and Methods

Experienced certified orthoptists photographed the forehead and eyes under the same room conditions, using a digital single-lens camera (CD-15CPX, Mitutoyo, Kanagawa, Japan). The patient was asked to relax, and a photograph was taken with natural open eyes. The following intraoperative variables were defined and evaluated:

1. Marginal reflex distance 1 (MRD_1_) and marginal reflex distance 2 (MRD_2_), corresponding to the distances between the corneal light reflex in the pupillary center and the margins of the upper and lower eyelids, respectively, when the eye is held in the primary position (Fig. 2a).
2. Inferior half area (IHA), corresponding to the area between the lower eyelid margin and below the baseline defined as the line connecting the medial and lateral corners (Fig. 2b).
3. Eyelash angular direction (EAD), corresponding to the tangential angle between the baseline and the lower eyelashes. The EAD was measured in the medial 1/6, central 3/6, and lateral 5/6 portions of the baseline (Fig. 2c) [8].
4. Side-view angle (SVA), corresponding to the average of the maximum and minimum angles between the eyelashes and a tangential line to the cornea (Fig. 2d) [6].

All patients received frontal view photographs for measuring MRD1, MRD2, IHA, and EAD at preoperative and last visit (9 months after the surgery). Lateral view photographs for measuring SVA were taken at preoperative and each postoperative visit (1 month, 3 months, 6 months, and 9 months after the surgery). Each factor was analyzed in digital photographs from front and side views taken during primary gaze and in a neutral head position. MRD_1_, MRD_2_, and IHA were standardized using by assuming a horizontal corneal diameter of 11 mm based on the findings of Terai et al. [9]. All of the measurement were performed twice by blinded observers (L.S.J. and L.M.S) using Image J software (NIH, Bethesda, MD, USA). Intergrader reliability (к) between two graders was assessed with к value, which ranged from 0.91 to 0.95 in each value.

### Statistical analysis

Statistical analyses were performed using SPSS (version 17.0, SPSS, Chicago, IL, USA). The Shapiro-Wilk test was used to determine whether the data conformed to a parametric (Gaussian) or nonparametric (non-Gaussian) distribution. Student’s *t*-tests were used to check for group differences between epiblepharon patients and controls (patients undergoing strabismus surgery). Paired *t*-tests were used for comparisons between pre- and postoperative changes at 1 month, 3 months, 6 months, and 9 months. The criterion for statistical significance was set at *p* < 0.05.

## Results

This study included 118 lower eyelids of 59 epiblepharon patients (23 females and 36 males aged 7.07 ± 2.23 years, mean ± SD). The follow-up period ranged from 9 months to 16 months (11.75 ± 2.55 months). All of these patients exhibited contact between the eyelashes and the cornea, and surgery was performed for signs and symptoms of corneal irritation. There were no cases of postoperative recurrences or other adverse effects such as overcorrection or prominent lower eyelid creases during the follow-up period. The study also included 89 control (178 eyes) age-matched strabismus patients who had no eyelid anomaly (46 females and 43 males aged 7.58 ± 2.14 years).

**Fig. 3:**
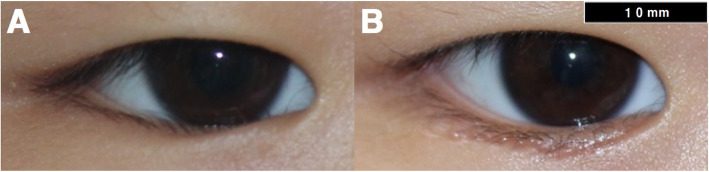
Change in MRD_1_ between preoperatively (**A**) and after epiblepharon surgery (**B**)

The preoperative MRD_1_, MRD_2_, and IHA in the epiblepharon patients were 1.92 ± 0.99 mm, 5.35 ± 0.92 mm, and 36.33 ± 9.78 mm^2^, respectively, which did not differ from the values in controls (*p* = 0.594, 0.095, and 0.187, respectively). After surgery, MRD_1_ increased to 2.50 ± 0.93 mm and IHA increased to 43.06 ± 10.57 mm^2^ (Fig. 3), whereas the postoperative MRD_2_ (5.33 ± 0.67 mm) had not changed compared to the preoperative value (*p* = 0.934) (Table 1).

**Table 1:** Comparison of eye features in controls and epiblepharon patients between pre- and postoperatively. MRD_1_, marginal reflex distance 1; MRD_2_, marginal reflex distance 2; IHA, inferior half area

	Controls	Epiblepharon patients		p^*^	p^**^
		preoperative	postoperative		
MRD_**1**_ (mm)	1.97 ± 1.02	1.92 ± 0.99	2.50 ± 0.93	0.594	**0.001**
MRD_2_ (mm)	5.52 ± 0.88	5.35 ± 0.92	5.33 ± 0.67	0.095	0.934
MRD_1_ + MRD_2_ (mm)	7.49 ± 1.17	7.26 ± 0.97	7.83 ± 1.05	0.053	**0.001**
IHA (mm^2^)	38.05 ± 10.71	36.33 ± 9.78	43.06 ± 10.57	0.187	**0.001**

Data are mean ± SD values. Significant *p* values are indicated in boldface

^*^between control and preoperative, ^**^between pre- and postoperative

**Fig. 4:**
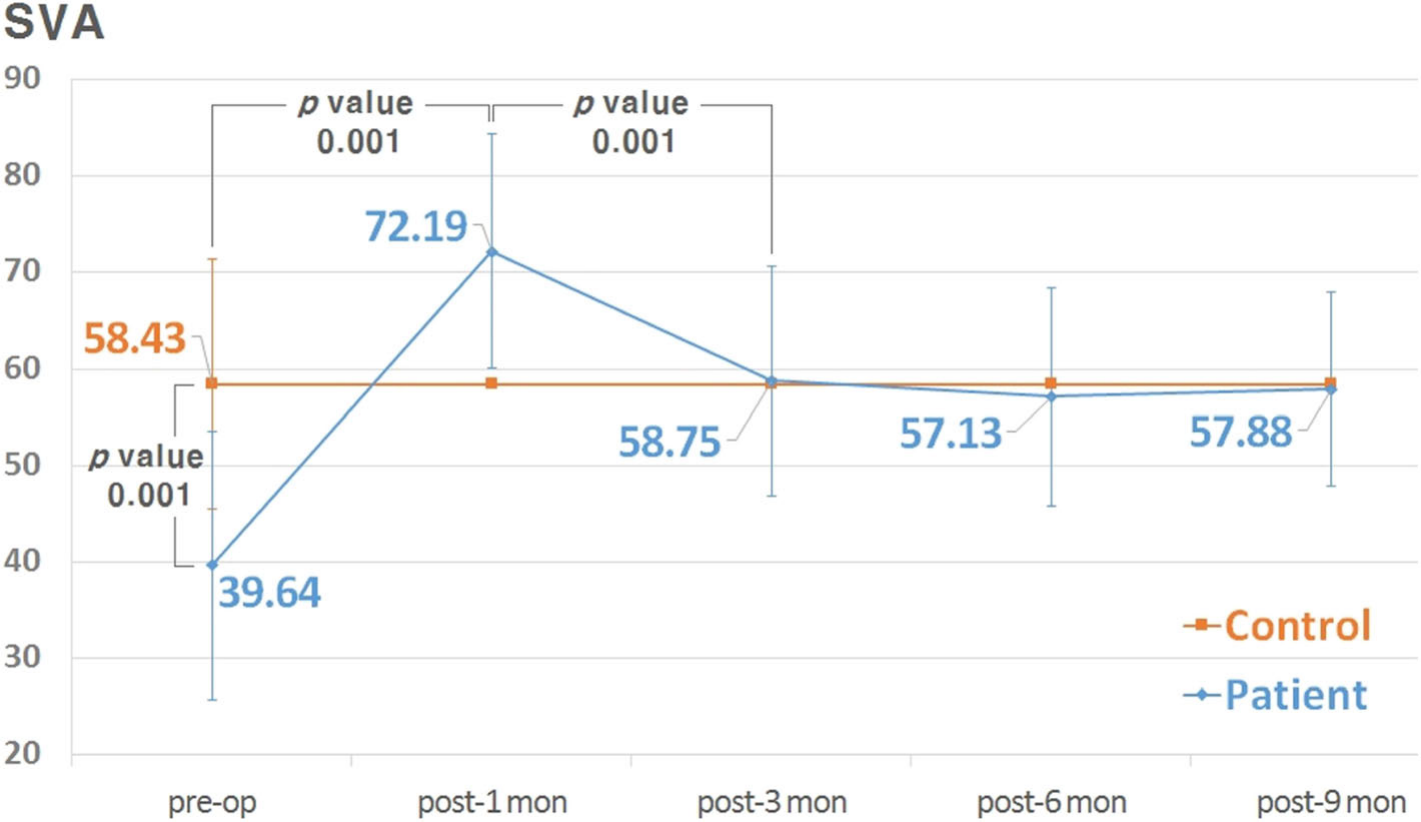
Comparison of SVA values between controls and epiblepharon patients pre- and postoperatively

**Fig. 5:**
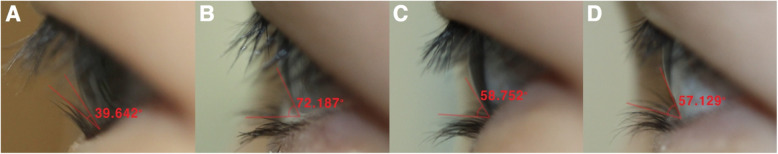
Photographs of a epiblepharon patient before and after surgery. **A** Preoperative SVA. **B** Postoperative SVA after 1 month was widened compared with preoperatively. **C** After 3 months, when it had narrowed again. **D** After 6 months, with no significant difference compared to after 3 months

The preoperative medial and central EAD in the epiblepharon patients were 92.45 ± 20.21 mm, 128.46 ± 23.49 mm, respectively, which were significantly larger than the values in controls (*p* = 0.016 and 0.001, respectively) (Table 2). The medial EAD was 92.45 ± 20.21° preoperatively and changed to 79.43 ± 23.31° after surgery (*p* = 0.004), while there were no significant changes in the central and lateral EADs (*p* = 0.535 and 0.093, respectively) (Table 2). The preoperative SVA was much narrower in the epiblepharon patients (39.64 ± 13.97°) than in the controls (58.43 ± 14.56°, *p* = 0.001). Postoperatively the angle changed to 72.19 ± 12.69°, 58.75 ± 12.72°, 57.13 ± 13.11°, and 57.88 ± 12.51° after 1 month, 3 months, 6 months, and 9 months, respectively, to approach the value in the controls (mean = 58.43°) (Figs. 4 and 5).

**Table 2:** Comparison of eyelash angular direction (EAD) in controls and epiblepharon patients between pre- and postoperatively

EAD	Controls	Patients		P^*^	P^**^
		preoperative	postoperative		
Medial 1/6	85.20 ± 28.03	92.45 ± 20.21	79.43 ± 23.31	**0.016**	**0.004**
Central 3/6	112.52 ± 42.79	128.46 ± 23.49	131.66 ± 36.85	**0.001**	0.535
Lateral 5/6	158.66 ± 10.02	160.36 ± 8.10	162.53 ± 5.74	0.121	0.093

Data indicated as in Table 1

Measurement unit (degree)

*between control and preoperative, **between pre- and postoperative

## Discussion

This study investigated the morphology of eyelid changes after epiblepharon surgery. Before performing the surgery, the parents had brought their children to hospital due to corneal irritation and related symptoms such as tearing and rubbing their eyes. There were improvements not only in the corneal irritation symptoms but also in the cosmetic eye widening. Thus, we selected 59 patients for whom reliable data were available, and analyzed the changes before and after surgery based on frontal and lateral perspectives.

In front views the medial EAD had changed the most significantly postoperatively (from 92.45° to 79.43°), while the central and lateral EADs were virtually unchanged (Table 2). This means that the medial portion plays the biggest role in postoperative changes; in other words, eyelid inversion and eyelash–cornea contact occur mainly in the medial portion, which is consistent with Kim et al. finding that lesions involving contact by lower eyelashes occurred mostly in the medial portion (58.6%), followed by central (37.9%) and lateral (3.4%) portions [10, 11].

A narrow SVA is the physical feature that most affects contact between the eyelashes and the cornea, and so widening this angle (by everting the eyelashes) is the main focus of surgery. In the present study the mean SVA had widened from 39.64° to 72.19° at 1 month after surgery, but then it had decreased to 58.75° at the 3-month follow-up. However, after then there was no more significant change, with the mean SVA stabilizing to 57.13° at 6 months and 57.88° at 9 months (Figs. 4 and 5). This stabilized value was consistent with that in the controls (58.43°, *p* = 0.466), indicating that surgery was successful without side effects such as eyelid retraction or ectropion.

IHA unexpectedly did no differ significantly between the epiblepharon patients preoperatively and the controls (*p* = 0.187) (Table 1). Epiblepharon is conventionally characterized by an extra fold of skin that directs the eyelashes into a vertical position, where they may contact the globe of the eye. However, our findings for IHA suggest that the cutaneous redundancy does not cover the eyes more than usual in Asians. This result is consistent with the study of Kakizaki et al. on the effect of redundant skin on eyelash inversion [12], in which redundant skin of the lower eyelid was not the main cause of eyelash inversion. The eyelash inversion did not improve despite a decrease in the cutaneous redundancy, which those authors attributed to other factors such as failure of the retractor-skin connection or hypertrophy of the orbicularis oculi muscle (OOM). IHA increased after surgery in the present study, resulting in cosmetic change, while MRD_2_ did not change (Fig. 3). This means that the increase in IHA mainly occurred in the medial rather than the central portion. This is consistent with change in EAD being most significant in the medial portion.

Another interesting finding is the change in MRD_1_ after lower epiblepharon surgery. While MRD_2_ remained unchanged, MRD_1_ increased from 1.92 ± 0.99 mm to 2.50 ± 0.93 mm (Fig. 3). Even when the lower part of the eye was incised, the increase occurred in the upper part of the eye. Previous studies have demonstrated that eyelid morphology could be changed by the surgeries remote from the eyelid, such as orbital decompression and strabismus surgery in thyroid eye diseases [13, 14]. Also, the reverse effect on the eyelid position has also been noted to change after ptosis repair [15].

We hypothesize that this was due to a tension change in OOM. Chen et al. reported that the cilial entropion of epiblepharon patients is related to the tension imbalance in OOM. In their study, the key point of epiblepharon repair is to release the tension of OOM, which rides over the eyelid margin [16]. In addition, Lee and Denise reported that the levator and frontalis muscles act as antagonists on OOM [17]. Therefore, due to the tension releasing of OOM after surgery, the role of the antagonists is relatively strengthened and the margin of the upper eyelid will be pulled up. Our opinion is that these tension change can contribute the increase in MRD_1_. Another possible mechanism for increasing MRD_1_ is a decrease in blinking. Kim et al. reported that frequent blinking is one of the chief complaints in epiblepharon [11]. If this blinking causes constant frowning, it can be thought that a postoperative improvement in frowning will increase the opening of the eye. Further, the assumption that this eye widening is associated with MRD_1_ is strengthened by Nathan reporting that blinking is almost solely due to the upper eyelid, with the lower eyelid remaining stationary [18]. In other words, when blinking decreases postoperatively, the change could occur mainly in the upper eyelid.

The limitations of this study are that there is no consideration of severity of epiblepharon. Time point of the postoperative result is not specified in MRD, IHA, and EAD. In addition, the data were confined to Asian children and interpreted over a narrow range without race or age analysis. It is well known that there are differences in the craniofacial anatomy (including of the eyelid) between Caucasians, Africans, and other races [19]. Nevertheless, it is meaningful to have a morphological basis for epiblepharon surgery in Asian children, especially since they account for a large proportion of such patients. However, future studies should investigate racial differences in the morphological changes after epiblepharon surgery.

In summary, this study performed a morphological analysis of epiblepharon patients before and after surgery. The symptoms experienced by epiblepharon patients usually occur in the medial eyelashes, but are postoperatively alleviated with the increase in SVA. However, since SVA was normalized through a little renarrowing between 1 month and 3 months, follow-up observations are required for at least 3 months to properly observe the surgical results. Also, epiblepharon patients experience appearance changes with increases in IHA and MRD_1._ Some parents will report that their children’s eyes have changed (e.g., looking bigger) after epiblepharon surgery. This study provides an objective basis for addressing parental concerns and answering their questions during presurgery counseling, such as about whether the appearance of their children’s eyes will change after epiblepharon surgery.

## Data Availability

The datasets generated during and analyzed during the current study are not publicly available due to personally identifiable data but are available from the corresponding author on reasonable request.

## Abbreviations

EAD: Eyelash angular direction
IHA: Inferior half area
MRD_1_: Marginal reflex distance 1
MRD_2_: Marginal reflex distance 2
OOM: Orbicularis oculi muscle
SVA: Side-view angle
